# S1-3 Creating Active Schools

**DOI:** 10.1093/eurpub/ckad133.006

**Published:** 2023-09-11

**Authors:** Andy Daly-Smith

**Affiliations:** Bradford University

## Abstract

**Purpose:**

The Creating Active Schools Framework was co-developed in June 2019 by 50 national and international stakeholders (teachers, headteachers, active school coordinators, Public Health leads, Active Partnership school leads, UK and international researchers, national sport and education organisations, members of the local delivery pilots). The co-developed CAS programme takes a professional development approach to support schools to review current provision and make evidence-informed decisions to address school policy, environments, and stakeholder behaviour to improve opportunities for children to be physically active (https://ijbnpa.biomedcentral.com/articles/10.1186/s12966-020-0917-z).

**Project description:**

Since conception, the framework has been adopted by practice and has currently scaled to work across 18 localities in England and over 200 schools. As part of this journey, the development of the online profiling tool (https://www.creatingactiveschools.org), a central piece of the intervention approach that places professional development for physical activity at the heart of the school community will be shared. Key learning on drawing partners and stakeholders from across, policy, research and practice together will be provided as well as an overview of the planned wider rollout of CAS (adding a further 20 localities and 200 schools from Sept 2023). Plans for the interdisciplinary evaluation of the implementation of CAS at scale and the cost(effectiveness) of the programme under naturalistic conditions will be provided.

**Conclusions:**

CAS, the UK’s first large-scale whole school physical activity programme, is uniquely placed to provide much-needed evidence to inform government investment and educational reform on how to scale health-based interventions under real-word conditions.

